# An introduction to MyBFF@school, a school-based childhood obesity intervention program: a cluster randomized controlled trial

**DOI:** 10.1186/s12889-025-21382-7

**Published:** 2025-01-14

**Authors:** Abdul Halim Mokhtar, Zahari Ishak, Fuziah Md. Zain, Rusidah Selamat, Abqariyah Yahya, Muhammad Yazid Jalaludin

**Affiliations:** 1 Department of Sports Medicine, Faculty of Medicine, Universiti Malaya, Kuala Lumpur, Wilayah Persekutuan Kuala Lumpur 50603 Malaysia; 2 Faculty for Sports and Exercise Science, Universiti Malaya, Kuala Lumpur, Wilayah Persekutuan Kuala Lumpur 50603 Malaysia; 3 FOSSLA, UCSI University, Kuala Lumpur, Wilayah Persekutuan Kuala Lumpur 56000 Malaysia; 4 Department of Pediatrics, Putrajaya Hospital, Ministry of Health, Jalan P9, Federal Government Administrative Centre Presint 7, Wilayah Persekutuan Putrajaya, Putrajaya, 62250 Malaysia; 5 Nutrition Division, Ministry of Health, Level 1, Block E3, Complex E, Federal Government Administrative Centre, Wilayah Persekutuan Putrajaya, Putrajaya, 62590 Malaysia; 6 Department of Social and Preventive Medicine, Faculty of Medicine, Universiti Malaya, Kuala Lumpur, Wilayah Persekutuan Kuala Lumpur, 50603 Malaysia; 7 Department of Pediatrics, Faculty of Medicine, Universiti Malaya, Kuala Lumpur, Wilayah Persekutuan Kuala Lumpur, 50603 Malaysia

**Keywords:** Obesity, Overweight, Schoolchildren, Children, Adolescent, Intervention

## Abstract

Obesity trend among Malaysian children is on the rise. Noting that the tendency for them to grow into obese adults and the relationship of obesity to many non-communicable diseases, the My Body is Fit and Fabulous at School (MyBFF@school program) was designed to combat obesity among the schoolchildren. The program was piloted in 2014 in Putrajaya, Malaysia. There were several challenges during the pilot study which included strain in manpower, limited variation of physical activity, nutrition, and psychology modules, time-constraint after school hours, co-curriculum marks, contamination effect, and school selection. The main MyBFF@school in 2016 addressed the challenges and improvised the design which were elaborated in subsequent articles in this supplement. This cluster randomized controlled trial was conducted in three states; Federal Territory of Kuala Lumpur, Selangor and Negeri Sembilan in 23 primary and 15 secondary schools were selected through proportionate random sampling. The MyBFF@school intervention package consisted of physical activity, nutrition and psychology components were carried out for six months. Data were collected at baseline, mid (month-3) and end (month-6) of the study period. The effects of the program on body composition, clinical, physical fitness, nutrition, and psychology were assessed in primary schoolchildren aged 9 to 11 years old (children age group) and secondary schoolchildren (adolescent) aged 13 to 16 years old. The prevalence of overweight and obesity at screening (N=22,816) were 29.4% in primary and 26.8% in secondary schoolchildren. Outcomes of the trial is presented in this supplement. In summary, the MyBFF@school program is a school-based intervention for overweight and obese children and adolescent. It is a combination of physical activity, nutrition and psychology components. We present in this supplement, the rationale, methodology and the outcomes of this randomized control trial of the MyBFF@school program.

## Background

The obesity trend across the world is increasing at an alarming rate [1]. More than 1.9 billion (39%) adults 18 years and above were overweight, whereas over 650 million (13%) were obese as reported by World Health Organization (WHO) [2]. Data from WHO also showed that approximately 170 million children aged less than 18 years old are currently prone to overweight. As for children aged less than 5 years old, the trend of overweight and obese was reported to increase from 32 million globally in 1990 to 41 million in 2016 [3]. In addition, the prevalence of overweight and obesity among children in developing countries is 30% higher than in developed countries [4].

In Malaysia, the National Health and Morbidity Survey (NHMS) 2019 found that the national prevalence of overweight and obese children aged 10–17 years was 29.8% (14.2% of girls and 15.7% of boys) [5]. Looking at the trend of obesity alone, the prevalence of obesity for children aged below 18 years old increased from 11.9% in 2015 [6] to 14.8% in 2019 [5]. According to the Global Obesity Observatory, the prevalence of overweight and obesity in the neighboring countries of Malaysia were: Singapore, 13% (2017); Indonesia, 14.8% (2015); Thailand, 16.3% (2016); Philippines, 9.1% (2015); Vietnam 22.5% (2018); Myanmar, 7.6% (2016); Laos, 11% (2015); Brunei, 35.2% (2014); Timor-Leste, 4.4% (2015) and Cambodia, 3.7% (2013) [7]. This made Malaysia as the second highest with childhood obesity prevalence in South East Asia region. Childhood obesity can track into adulthood [8]. As obesity is related to a number of non-communicable diseases [9], certain types of cancer [10] and mental health issues [11], addressing this early may minimize the risk [12]. In fact, the Academy of Medical Sciences reported approximately 73% of annual deaths in Malaysia is due to non-communicable diseases which are highly associated with obesity [13]. This raises concern and urgency to intervene in childhood obesity.

There are a number of obesity intervention programs worldwide [14–20]. The results however, are mixed. One of the successful interventions includes peer educators initiative adopted from Social Cognitive Theory (SCT) in which schoolchildren deliver educational material to their counterparts. This initiative resulted in positive behavioral changes both among the schoolchildren and the peer educators, as well as enhancing their awareness on obesity [21]. Similar approach was proven as an effective tool in promoting good health behavior and attitudes [22]. There was also a significant increase in physical activity level due to the implementation of integrated interventions which took up both obesity and eating disorder issue [21]. In contrast, some programs were unsuccessful due to short intervention period [23], methodological limitations such as lack of quantitative assessment, and the use of BMI alone as an indicator for obesity [24, 25]. School-based program is noted to be one major key to a successful intervention. This is further concurred by several international guidelines on childhood obesity intervention [26–28].

The MyBFF@school intervention program was designed to combat overweight and obesity in Malaysian schoolchildren which consisted of multi-components i.e. physical activity, nutrition and psychology. A pilot study was conducted in Putrajaya, Malaysia (MyBFF@school 2014) with a total of 425 participants. A total of 237 schoolchildren were from 11 primary schools (aged 9 to 11 years) and 188 schoolchildren from secondary schools (aged 13 to 16 years) [29]. The program was run just after school hours, so as not to interrupt the existing school curriculum. From the Working Group Discussions (WGD) between the research assistants (RA), and the teachers as well as with the students, a number of challenges encountered during the pilot study were tackled when the main MyBFF@school study was conducted in 2016 (MyBFF@school 2016).

Firstly, the importance of getting a specific personnel to run the program at schools was addressed. School teachers could not be relied on as they were tied up with their own school responsibilities. Furthermore, the program was conducted after school hours that would further stretch the teachers’ working time. Apart from that, there was a need for specific person assigned to conduct and monitor the program to ensure every module from each component are followed as per designed.

During the pilot study, football was the only game played as small-sided games (SSG) of the physical activity component. The schoolchildren enjoyed the game, but requested more types of games to be included e.g. hand-games. Therefore, hand-games and fun-games were added in the SSG while maintaining the football games in MyBFF@school 2016 study. This enriched and increased the variety of physical activity and could contribute to the enjoyment and compliance of the schoolchildren.

Another challenge faced was a high attrition rate (about 50%). Among the main reasons was that the program was conducted after school hours. Quite a handsome number of schoolchildren had other additional classes to attend after their school hours, including the religious school (non-mandatory but popular local second school). On top of this, the schoolchildren also faced logistic challenges as their transport schedule was interrupted due to the after-school hours MyBFF@school 2014 program. Another contributing factor was, unlike other co-curriculum activity, MyBFF@school program did not carry any supportive points for the schoolchildren, hence seen as less-important by the parents. These issues were rectified by conducting the MyBFF@school 2016 program during school hours and getting co-curriculum point for the program, i.e. like any other school co-curriculum activity. Less attrition rate was expected as there would be minimal interference in their daily activities besides satisfying the co-curriculum requirement of the school.

Another shortcoming was that the nutrition and psychology components, being a class-based modules, were less attractive to the schoolchildren. The classes were improvised by having more practical, playful and interactive sessions. The nutrition component added interesting activities, e.g. teaching children to cook healthy food in ‘Let’s Cook’ session and outdoor activities e.g. ‘Smart Shopping’. Whilst more ‘tools’ are used, e.g. plaster, clay and role-play games in psychology module.

In terms of schools’ location, all schools during MyBFF@school 2014, were selected from the same vicinity i.e. Putrajaya. Putrajaya schools are urban type with the majority population of Malay ethnicity. The possibility of contamination effect between intervention and control schools could not be avoided due to their close vicinity. This problem was corrected by increasing the number of schools from a larger area (three states in Peninsular of Malaysia) and added vernacular schools too, hence reducing the possibility of being too close avoiding contamination effect and increased the diversity in ethnicity. Finally, the pilot study’s research design was quasi-experimental. The study design was later consolidated to a cluster randomized controlled trial for the current MyBFF@school study.

Having addressed the limitation aforementioned, MyBFF@school set forth in 2016. The three states mentioned above are the Federal Territory of Kuala Lumpur, Selangor and Negeri Sembilan. The target groups of this study were primary schoolchildren aged 9 to 11 years (children age group) and secondary schoolchildren aged 13 to 16 years old (adolescent age group) with BMI *z*-score of > 1 SD. In essence, 1196 primary and 416 secondary schools were screened i.e. a total of 22,816 schoolchildren. Twenty-three primary and 15 secondary schools with a total of 2438 (n=1397-primary and n=1041-secondary) schoolchildren were finally selected for the study through proportionate random sampling. The MyBFF@school program intervention package maintained the three major components, but improved them i.e. by increasing the types of games in physical activity and more interactive sessions of nutrition and psychology. The study was carried out for six months i.e. from mid-February until mid-August, whereby the intervention schools followed the MyBFF@school program, while the control schools followed the existing standard school program. Data were collected at baseline, mid (month-3) and end (month-6) of the study period. The effects of MyBFF@school program on body composition, clinical, physical fitness, nutrition, and psychology were investigated. The methodology and various outcomes are presented in this supplement.

The protocol paper described the rationale, methodology and baseline findings [30]. Previous interventions on childhood obesity were discussed, heeding on what worked and not and their limitations. The paper described the MyBFF@school intervention package and presented the baseline findings that gave an overview of the studied population including age, gender, ethnicity, school location (rural and urban), body composition and the screening. The high prevalence of overweight and obesity i.e. 29.4% in primary and 26.8% in secondary schoolchildren justified the urgency for obesity intervention in both primary and secondary schoolchildren.

The ensuing articles in this supplement described the anthropometric, cardiometabolic, cardiorespiratory fitness, nutrition and psychological outcomes of the MyBFF@school study. In summary, the MyBFF@school program is a school-based intervention for overweight and obese children and adolescent. It is a combination of physical activity, nutrition and psychology components. We present in this supplement, the rationale, methodology and the outcomes of this randomized control trial of the MyBFF@school program.

## Data Availability

All relevant data are within the paper.

## Abbreviations

BMI: Body mass index
HDL-C: High-density lipoprotein cholesterol
HRQOL: Health-related quality of life
HOMA-IR: Homeostatic model assessment of insulin resistance
MyBFF@school: My Body is Fit and Fabulous
NHMS: National Health and Morbidity Survey
PBF: Percentage body fat
RA: Research assistant
SMM: Skeletal muscle mass
SSG: Small-sided games
SCT: Social Cognitive Theory
TG:HDL-C ratio: Triglycerides to high-density lipoprotein cholesterol ratio
YSR: Youth-Self report
WC: Waist circumference
WGD: Working group discussion
WHO: World Health Organization
